# Atorvastatin for reduction of 28-day mortality in hospitalized COVID-19 patients: study protocol for a randomized, double-blinded, placebo-controlled, clinical trial

**DOI:** 10.1186/s13063-022-06619-9

**Published:** 2022-08-08

**Authors:** Moataz Maher Emara, Neamat Hamdy Elsawy, Kholoud M. Abdelaaty, Amal Salah Elhamaky, Naglaa Hamdi Eltahan

**Affiliations:** 1 Department of Anesthesiology and Intensive Care and Pain Medicine, Mansoura University Faculty of Medicine, 60 Elgomhoria St, Mansoura, Egypt; 2 Fowa Central Hospital, Ministry of Health and Population, Cairo, Egypt; 3 Specialized Medical Hospital, Mansoura University, Mansoura, Egypt; 4 Mansoura Specialized Hospital, Ministry of Health and Population, Cairo, Egypt; 5 Sherbin Central Hospital, Ministry of Health and Population, Cairo, Egypt

**Keywords:** COVID-19, SARS-CoV-2, Coronavirus, Statins, Hmg coa, Atorvastatin, Mortality, Randomized controlled trial

## Abstract

**Background:**

Although mass vaccination has reduced the severity of COVID-19, mortality is still high among hospitalized patients. Being a sepsis-like disease, an anti-inflammatory drug as atorvastatin would reduce mortality and severity in COVID-19.

**Methods:**

We designed a randomized clinical trial that recruited 220 COVID-19 patients admitted in the COVID-19 isolation hospital at Mansoura University, Egypt. One hundred ten cases were assigned to receive 40 mg atorvastatin once daily for 28 days, and 110 were assigned to receive placebo. Delta Pharm company supported the study with the drug and the placebo, which mimics the drug as regards the drug package, the tablet color, consistency, and size. All patients received the standard treatment as per the hospital protocol. The Institutional Review Board approval and the informed consent from all participants were obtained.

The primary outcome is the 28-day all-cause mortality. Additionally, we will collect the in-hospital mortality, the need for mechanical ventilation, time to clinical improvement, in-hospital thrombo-embolic events, acute kidney injury, and the hospital and the intensive care duration of stay. We plan to follow the patients up for 6 months for reporting mortality and long-term neurological, psychological, and respiratory consequences.

We will report the un-adjusted 28-mortality using *χ*^*2*^. Then, we will report the adjusted odds ratio with a pre-planned multiple logistic regression model. We will report our results using the point estimate and the 95% confidence interval and the *P*-value.

**Discussion:**

The additional issue that we would like to discuss is the added workload on the clinicians and the allied healthcare workers who performed research at the time of the pandemic. Therefore, doing research at the pandemic era was, indeed, challenging.

**Trial registration:**

The study was registered at the Clinical Trial Registry (NCT04952350) on July 1st, 2021.

**Supplementary Information:**

The online version contains supplementary material available at 10.1186/s13063-022-06619-9.

## Administrative information

Note: the numbers in curly brackets in this protocol refer to SPIRIT checklist item numbers. The order of the items has been modified to group similar items (see http://www.equator-network.org/reporting-guidelines/spirit-2013-statement-defining-standard-protocol-items-for-clinical-trials/).

**Table:** 

Title {1}	Atorvastatin for reduction of 28-day Mortality in Hospitalized COVID-19 Patients: A Randomized, Double-blinded, Placebo-Controlled, Clinical Trial
Trial registration {2a and 2b}.	NCT04952350 at ClinicalTrials.gov https://clinicaltrials.gov/ct2/show/NCT04952350
Protocol version {3}	V03. 1st Jul 2021
Funding {4}	The company that will provide the research group with the drug and the placebo will not support additional funds or interests. The investigators declare no relevant conflict of interest.
Author details {5a}	1. Moataz Maher Emara, MD, EDAIC Mansoura University, Faculty of Medicine, Egypt 2. Neamat Hamdy Elsawy, Pharm D Fowa Central Hospital, Ministry of Health and Population, Egypt 3. Kholoud M. Abdelaaty, BCPS Specialized Medical Hospital, Mansoura university, Egypt 4. Amal Salah Elhamaky Mansoura Specialized Hospital, Ministry of Health and Population, Egypt 5. Naglaa Hamdi Eltahan Sherbin central hospital, Ministry of Health and Population, Egypt
Name and contact information for the trial sponsor {5b}	Mansoura University Address: Elgomhouria St., Mansoura City, Egypt 35516 Email: mua@mans.edu.eg Tel. +20 (50) 2383781 / +20 (50) 2397055 / +20 (50) 2397054, FAX: +20 (50) 239733 / +20 (50) 2397900 Website: http://www.mans.edu.eg
Role of sponsor {5c}	The sponsor only supports the research conducted through institutional resources

## Introduction

### Background and rationale {6a}

COVID-19 has been a major global health problem. Despite mass vaccination, waves of epidemics are still striking. SARS-CoV-2 has infected more than 265 million cases all over the world. By December 5, 2021, COVID-19 caused more than 5.2 million deaths worldwide and approximately 20,919 deaths in Egypt [1]. COVID-19 increases the risk of pneumonia, cardiovascular, and thromboembolic events [2].

COVID-19 implies an aggressive immune reaction—the cytokine storm [3]. Elevated cytokines—as interleukin-6 (IL-6)—activate the nuclear factor kappa B (NF-κB) pathway, which causes sepsis, capillary damage, acute pulmonary injury, severe acute respiratory distress (ARDS), multi-organ damage, and death [3, 4]. COVID-19 survivors suffered the post-COVID syndrome, which includes pulmonary, cardiovascular, renal, and neuropsychiatric consequences. These long-term effects may result from a direct viral infection, systemic inflammation, neuroinflammation, microvascular thrombosis, and neurodegeneration [5].

β-Hydroxy β-methyl glutaryl-CoA (HMG-CoA) drugs—statins—are lipid-lowering drugs with pleiotropic effects. Statins might benefit COVID-19 patients due to their anti-inflammatory and antioxidant effects: (1) bind to the SARS-CoV-2 main protease and prevent invasion of the host cells [6]; (2) activate autophagy and regulate SARS-CoV-2 virus degradation or replication, which may reduce viral load; (3) modulate the immune response by blocking the NF-κB pathway and NLRP3 inflammasomes, so theoretically decrease the cytokine storm in COVID-19 [6]; and (4) improve the endothelial function, which participates in the pathogenesis of the COVID-19 [7].

Statins propose antithrombotic and anticoagulant effects. In a chemical-induced venous thrombosis in a murine model, atorvastatin enhanced the vein thrombus resolution [8]. In addition, using statins was associated with a lower risk of recurrent pulmonary embolism (PE) by 50 % [7].

In influenza pneumonia, studies suggest the survival benefits of statins. One study found that statins were associated with a 0.59 reduction in the odds of death before or during hospitalization [9]. Another study found a hazard ratio of death of 0.41 with a matched sample [10]. Besides, simvastatin significantly improved the 28-day survival in hyperinflammatory ARDS [11]. Speaking of COVID, statins reduced mortality by almost half with an adjusted hazard ratio (HR) of 0.58 or slowed the progression of death [12, 13].

As regards the potential neuroprotective effects of statins, they reduced the risk of Alzheimer’s disease and related dementia with HR 0.54 (0.32–0.91) and 2.45 (0.69–8.68) compared to non-statin users [14]. Statins also have neuroprotective benefits by regulating cholesterol metabolism in the brain [15].

Consequently, we designed a randomized clinical trial to investigate the role of atorvastatin in hospitalized (severe and critical cases) COVID-19 patients on the 28-day mortality and the 6-month long-term consequences. We hypothesized that atorvastatin 40 mg once daily for 28 days would reduce the 28-day all-cause mortality in adult hospitalized COVID-19 patients.

## Objectives {7}

### The study hypothesis

Atorvastatin 40 mg per day for 28 days would reduce the 28-day all-cause mortality in adult hospitalized (severe and critical) COVID-19 patients.

### The objective of the study

The objective of the study is to test the efficacy of atorvastatin 40 mg per day in reducing 28-day mortality and the 6-month consequences in hospitalized patients with severe and critical COVID19.

## Methods: participants, interventions, and outcomes {8}

We wrote the protocol according to the recommendations of the Standard Protocol Items for clinical Trials (SPIRIT 2013) guidelines [16].

The study design is as follows: double-blind randomized (1:1) placebo-controlled superiority trial.

### Study setting {9}

The study setting is as follows: Mansoura University COVID-19 Isolation Hospital, Mansoura city, Egypt

### Eligibility criteria {10}

#### Inclusion criteria

We included adult patients (≥18 years old) with severe or critical COVID-19 admitted to the COVID-19 Mansoura University Isolation Hospital. We included patients who are PCR-confirmed, clinically or radiologically diagnosed with COVID-19.

Cases are defined as severe or critical according to the WHO definition [17], where severe cases have the clinical signs of severe pneumonia and SpO_2_ < 90% on room air or RR > 30 breaths/min without any critical criteria, while critical cases have ARDS, or sepsis, septic shock or pulmonary embolism, acute coronary syndrome, or acute stroke.

#### Exclusion criteria

Exclusion criteria were chronic statin use, serum creatine kinase (CK) > 5 times the upper limit of normal (ULN), serum transaminases > 5 times ULN, acute hepatic failure, chronic liver disease (Child-Pugh Classification C), history of rhabdomyolysis or myopathies, severe renal impairment not receiving renal replacement therapy (estimated Cr cl< 30 ml/min ), pregnant and lactating women, patients who are expected to die within 48 h, or patients on chronic colchicine, cyclosporine, or ritonavir.

### Who will take informed consent? {26a}

We secured written informed consent from the patient or the legal representative if the patient was unable to give consent. The IRB of Mansoura University – Faculty of Medicine approved the protocol (Supplement S1).

One of the investigators conducted the consent process with the patient or the legal representative by signing two copies (one for the investigator and one for the patient). The consent was signed and dated by the conducting investigator and the consent provider.

Patients or their legal representatives were informed clearly that all patients have the same chances to receive the active treatment or the placebo.

We provided the informed consent form as a supplement to this protocol (Supplement S2).

### Additional consent provisions for collection and use of participant data and biological specimens {26b}

The patient consent discussed the patients’ publication of their anonymous data. The biological specimens were handled according to the institution’s protocol (Supplement S2).

### Interventions {6b}

#### Placebo

Patients in the placebo group received the standard treatment protocol according to the local guidelines. The placebo resembles the original drug regards the drug package, the tablet color, consistency, and size.

Atorvastatin has not proved its efficacy yet, so there is no harm in not receiving atorvastatin in COVID19 patients, according to the Declaration of Helsinki 2008, paragraph 32 [18].

#### Intervention {11a}

Patients were randomized to receive either placebo or enteral atorvastatin 40 mg/day for 28 days.

### Administration in unconscious or ventilated patients

The patients received the drug through a nasogastric tube.

### Criteria for discontinuing or modifying allocated interventions {11b}

The safety assessment included daily monitoring for adverse events; we checked CK level if the patient developed myalgia or unexplained weakness and stopped if CK > 10 times ULN, drug-induced hepatitis, or serum transaminases > 8 times ULN. Patients who received azole antifungals had a lower threshold for monitoring of liver injury and rhabdomyolysis.

### Strategies to improve adherence to interventions {11c}

Patients started the drug during hospitalization, and, on discharge, they received the estimated amount of their study drug and were educated to complete the 28-day treatment. Additionally, we contacted them by telephone (or WhatsApp) after their discharge to ensure adherence to treatment.

### Relevant concomitant care permitted or prohibited during the trial {11d}

All patients received the standard of care according to local hospital protocol. Antiviral treatment was allowed and recorded.

### Provisions for post-trial care {30}

We will conduct a 6-month follow-up via telephone and at the outpatient clinic. An interview with all participants will be face-to-face by trained physicians and asked to complete a series of questionnaires, including a self-reported symptom questionnaire. We will report a detailed 6-months follow-up data later.

### Outcomes {12}

#### Primary outcome

All-cause mortality will be recorded within 28 days after randomization. We will ascertain mortality by a telephone call or the national death index.

#### Secondary outcomes

A) *Early outcomes*
  1. Need for invasive mechanical ventilation
  2. Invasive mechanical ventilation and oxygen support duration (days)
  3. Time to clinical improvement (defined as 2-point reduction in the WHO disease ordinal progression scale [19] or discharge, whatever happens first)
  4. Serious adverse effect leading to drug discontinuation
  5. ICU and hospitalization length of stay (days)
  6. CRP on days 3,7, 14, and 28
  7. SOFA score on days 3, 7, 14, and 28
  8. COVID19 WHO disease progression scale on days 3, 7, 14, and 28
  9. Incidence of acute kidney injury (AKI), defined as an increase in Scr by ≥ 0.3 mg/dl in 48 h or increase in Scr by ≥ 50% in 7 days or oliguria for ≥ 6 h [20]
  10. In-hospital deep vein thrombosis or PE
  11. Mortality at hospital discharge

CRP, D-dimers, SOFA scores, WHO disease progression score, and AKI were evaluated only during the hospitalization period.

B) *Late outcomes*: (will be published in a separate report)
  1. 6-month all-cause mortality
  2. Long-term outcomes of 6 months post-acute COVID19 syndrome
    ▪ Pulmonary function tests (PFTs), high-resolution computed tomography of the chest, and incidence of cognitive impairment.
    ▪ Health-related quality of life (anxiety or depression) and re-hospitalization rates
    ▪ Extrapulmonary organ function (including eGFR, and ultrasonographic features of kidney, and liver)

### Participant timeline {13}

**Fig. 1:**
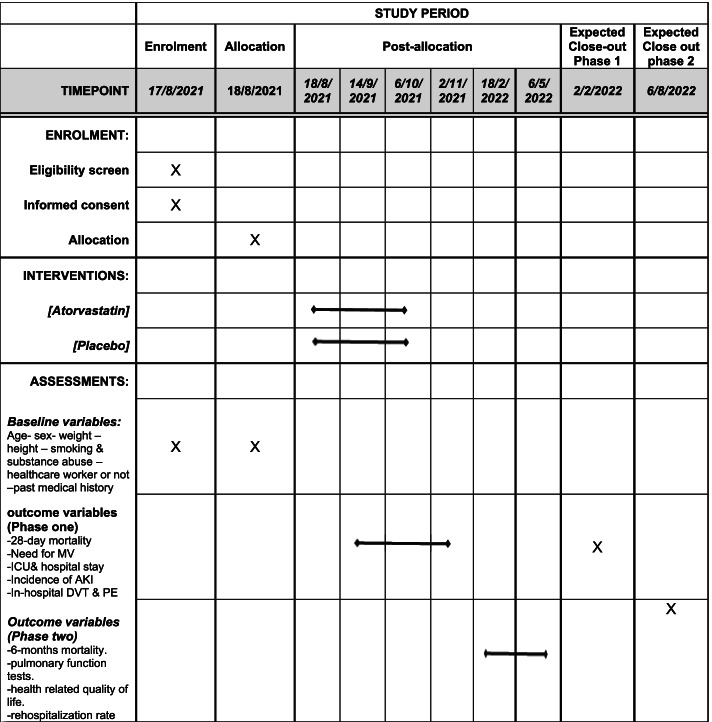
Schedule of enrolment, interventions, and assessments according to the SPIRIT template for the study timepoints

We started enrolment on August 17, 2021, and finished recruitment on October 6, 2021. The ascertainment of the primary outcome for the last enrolled patient was on November 2. Figure 1 shows the timeline of the study.

### Sample size {14}

A sample size of 97 patients in each group would achieve at least 80 % power to detect a risk difference of 0.2 (20%) in the 28-day all-cause mortality (primary outcome) between the null hypothesis (both proportions are 0.6) and the alternative hypothesis (the proportion in the non-statin group would be 0.4).

Based on two large studies on chronic use of statin in hospitalized COVID patients, the adjusted OR of mortality was 0.52 and 0.67, which corresponds to a risk reduction of 17–27 % in mortality when the mortality is 60% in the control group; therefore, we used the 20% reduction in our assumption of the sample size [21, 22].

We assumed a significance level (*α*) of 0.05 and used the chi-square test of independent proportions in MedCalc software.

To compensate for the estimated loss-to-follow-up, we increased the sample size to be 110 patients in each group.

The mortality in the control group (60%) was estimated from the average mortality in January, February, and March 2021 at the Mansoura University Isolation Hospital among all hospitalized patients.

### Recruitment {15}

According to the current admission rate at the Mansoura University – Isolation Hospital, the admission rate is 250 cases per month on average; our needed sample is 220 cases. We have already finished patient recruitment.

### Assignment of interventions: allocation

#### Sequence generation {16a}

A pharmacist, not involved in the study, created a randomization table (1:1) using computer-generated random permuted blocks (4, or 6 in each block).

#### Allocation concealment {16b}

The same pharmacist allocated patients to either group, without making the randomization table available to the study group. Randomization was conducted within 24 h after hospital admission after evaluation of the inclusion criteria. We started patient recruitment only after the registration of the study protocol on ClinicalTrial.gov.

Patients were randomized to receive either placebo or atorvastatin 40 mg/day orally for 28 days. All patients received the standard of care according to local hospital protocol. Antiviral treatment was allowed and reported.

#### Implementation {16c}

Randomization table was available only with the pharmacist who assigned each group for the intervention or the control.

### Assignment of interventions: blinding {17a}

Patients, caregivers, data collectors, and data analysts are blinded for the study group. The Delta Pharma company (Egypt) provided the atorvastatin and a similar placebo. The company did not—and will not—participate in any step of the study, including participant recruitment, data collection, data analysis, or results reporting. The company prepared a similar package of drug and placebo (labeled as A and B).

Even the pharmacist—involved in treatment allocation—will not know what the treatment group is, just A or B.

#### Procedure for unblinding if needed {17b}

Data Safety and Monitoring Board (DSMB) will be kept blinded for the assignment groups except if the termination of the study is an issue.

For emergency unmasking, one of the directors of Mansoura COVID-19 research council, who will not participate in the study, will have the true labels and the randomization table in a closed envelope. The envelope will be only opened if DSMB decided to unmask for a safety issue.

### Data collection and management

#### Plans for assessment and collection of outcomes {18a}

Our primary outcome, 28-day all-cause mortality, is highly objective. We ascertained mortality by a telephone call or by reviewing the national death index. Outcome assessors were trained to collect the COVID19 WHO disease progression score.

We added the definitions of the outcomes to the paper CRF to guide the outcome assessor while collecting data. Age and study durations were collected as dates (i.e., date of birth, date of admission, and date of discharge) to calculate the continuous variable on data cleaning before analysis.

Categorical outcomes were ascertained as continuous outcomes—as appropriate—and were categorized on the data analysis stage. The CRF—with the data and code dictionary—is available as a supplementary file (S3) to the protocol.

#### Plans to promote participant retention and complete follow-up {18b}

Patients started and almost received their intervention within the hospital. We educated patients and emphasized receiving their study drug after hospital discharge and contacted with telephones for follow-up. On the hospital discharge, patients received their expected doses of the assigned intervention.

#### Data management {19}

We collected the data in a paper case report form (CRF) and upload the data into an electronic CRF after 1–2 months of the patient enrolment. Paper CRF with the proposed coding was prepared **(**supplement S3), coding for missing data as 9999. Four investigators double-checked the data (2 investigators per 110 cases).

#### Confidentiality {27}

Patient confidentiality will be kept before, during, and after the study. Anonymized CRF papers were uploaded to electronic CRF. After the end of the study, the paper CRF will be kept in the hospital record room—only accessed by the research team after authorization of the IRB.

#### Plans for collection, laboratory evaluation, and storage of biological specimens for genetic or molecular analysis in this trial/future use {33}

We did not plan to keep the samples for further evaluation or future studies. Laboratory and biological specimens were manipulated according to the institution’s protocol.

### Statistical methods

#### Statistical methods for primary and secondary outcomes {20a}

The primary analysis will be based on the intention-to-treat strategy.

Categorical variables will be presented as proportion and percent. Continuous variables will be presented as mean (standard deviation) for parametric data or as a median (25th-75th percentile) for non-parametric data.

Chi-square test and *t*-test will be used to conduct a univariate analysis of demographic features correlated with study groups. We will report the 95% confidence interval and the *P*-value for our statistical tests.

In each group, we will compare the 28-day all-cause mortality rate (primary outcome) using the chi-square test with reporting the rate ratio and 95% confidence interval.

Statistical analysis will be achieved with SPSS, version 26. The level of statistical significance will be *p*-value ≤ 0.05.

#### Interim analyses {21b}

After recruitment of 25, 50, and 75% of the planned sample size, we performed interim analyses of the in-hospital mortality.

**Fig. 2:**
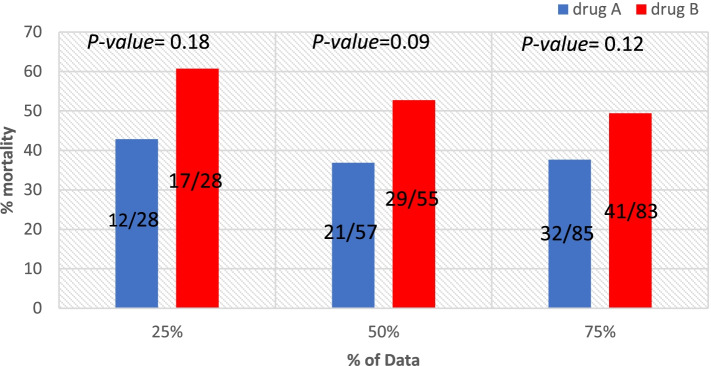
Interim analyses of the in-hospital mortality analyzed using *χ*^2^ at 25%, 50%, and 75% of patients, reporting the percent on the *Y*-axis and the number/total within the bars

The Data Safety Monitoring Board (DSMB) and the IRB members were blinded for the study allocation. Results of the interim analysis are presented in Fig. 2. They were timely reported and revised by the IRB and the DSMB.

#### Methods for additional analyses (e.g., subgroup analyses) {20b}

Subgroup analysis will be conducted for the following subset which will be selected 48 hours after recruitment:

- Patients with and without invasive mechanical ventilation
- Severe cases and critically ill cases

We will run a multiple logistic regression for the association between receiving atorvastatin and 28-day all-cause mortality, controlling for age, gender, the number of comorbidities (DM, HTN, IHD, AF, COPD), and COVID19 severity (severe or critical).

A multi-linear regression model will be used to assess the reduction in ventilation duration, controlling for the same risk factors in the logistic regression model.

#### Methods in analysis to handle protocol non-adherence and any statistical methods to handle missing data {20c}

We are planning to perform an intention-to-treat analysis, and we will manage missed data by analyzing the available data or by multiple imputations—reporting the method used.

#### Plans to give access to the full protocol, participant level-data and statistical code {31c}

We seek to publish the full protocol; besides, we have uploaded the protocol on the clinical trial registry. The CRF with the statistical code is provided as a supplement (supplement S3) to the protocol.

### Oversight and monitoring

#### Composition of the coordinating center and trial steering committee {5d}

The lead investigator is responsible for trial supervision, local organization, and identifying of the potential recruits. One chest physician and a clinical pharmacist conducted the informed consent process. The trial members met periodically online to oversee conduct and progress—at least at the time of evaluation of the interim analyses. In addition, we used WhatsApp for instantaneous communications—without circulating any of the patients’ data identifiers.

#### Composition of the data monitoring committee, its role and reporting structure {21a}

We had the COVID-19 scientific committee at Mansoura University as the DSMB. The members of the board have no direct role in the study. They monitor and evaluate the study conduct, interim analyses, and adverse events.

#### Adverse event reporting and harms {22}

We planned to report any in-hospital adverse events by the study investigators or the out-of-hospital adverse events by the patients. Patients have telephone contacts with WhatsApp for reporting any adverse events or discontinuation of the study drug. We did not receive any serious adverse events to report to the IRB or the DSMB.

#### Frequency and plans for auditing trial conduct {23}

We met at least for 3 times during the conduct of the study to view the interim analyses and to review the trial conduct. We contacted the IRB and the DSMB at each interim analysis stage to report the study progress.

#### Plans for communicating important protocol amendments to relevant parties (e.g., trial participants, ethical committees) {25}

We reported the interim analyses results to the IRB and the DSMB. Additionally, we reported the end of the patients’ recruitment to the IRB, DSMB, and updated the study status on the Clinical Trial registry.

#### Dissemination plans {31a}

We would report our results in a preprint server while aiming for publication in peer-review journals. We will report the early study outcomes, then the late 6-month outcomes in a separate later-on publication.

## Discussion

The additional issue that we would like to discuss is the added workload on the clinicians and the allied healthcare workers who performed research at the time of the pandemic. Therefore, doing research at the pandemic era was, indeed, challenging. At the beginning, the stakeholders did not give the researchers the needed space, however, we had some support at Mansoura University, and we could involve the COVID scientific committee to support the study.

## Trial status

This is the third version of the protocol approved by the IRB at Mansoura University, Faculty of Medicine. We started the study recruitment on August 18, 2021, and the patients’ recruitment end on October 6, 2021. Currently, we are collecting data for the long-term follow-up. Due to the pandemic workload, the investigators could not submit the protocol for publication at earlier stage.

## Supplementary Information

**Additional file 1: Supplement S1.** IRB approval

**Additional file 2: Supplement S2.** Informed Consent form (English Translation)

**Additional file 3: Supplement S3.** Case Report File (CRF)

## Data Availability

We will make the anonymous individual data available at reasonable request after publication. Patient confidentiality will be kept.

## Abbreviations

AF: Atrial fibrillation
AKI: Acute kidney injury
ARDS: Acute respiratory distress syndrome
CARDS: COVID-19 ARDS
CK: Creatine kinase
COPD: Chronic obstructive pulmonary disease
COVID-19: Coronavirus disease of 2019
CRF: Case report form
DM: Diabetes mellitus
DSMB: Data and Safety Monitoring Board
HMG-CoA: β-Hydroxy β-methylglutaryl-CoA
HTN: Hypertension
ICU: Intensive care unit
IHD: Ischemic heart disease
IL-6: Interleukin-6
IRB: Institutional research board
NF-κB: Nuclear factor kappa B
NLRP3: Nod-like receptor pyrin domain containing 3
PAI-1: Plasminogen activator inhibitor-1
PCR: Polymerase chain reaction
PE: Pulmonary embolism
PFTs: Pulmonary function tests
RR: Respiratory rate
SARS: Sever acute respiratory syndrome
SARS-COV-2: Severe acute respiratory syndrome - coronavirus 2
SOFA score: Sequential organ failure assessment
