# Supplementary material for: Atorvastatin for reduction of 28-day mortality in hospitalized COVID-19 patients: study protocol for a randomized, double-blinded, placebo-controlled, clinical trial
Source: Trials. 2022 Aug 8;23:636. doi: 10.1186/s13063-022-06619-9 (PMC9360729; doi:10.1186/s13063-022-06619-9)
Supplement: Supplementary file 2 — Additional file 2: Supplement S2. Informed Consent form (English Translation) [file 13063_2022_6619_MOESM2_ESM.docx]

**Informed Consent form (English Translation)**

**A randomized controlled trial to evaluate the effect of atorvastatin on mortality in adult hospitalized patients with COVID-19.**

**Participant identification number:……………..**

You are invited to participate in a medical research study on the effect of atorvastatin on mortality in adult hospitalized patients with COVID-19. We ask you to read this document carefully and ask any questions you may have before agreeing to be in the study.

1. **Purpose of the research:**

COVID-19 is a disease that was emerged in China in December 2019 and became a leading cause of death worldwide. It affected nearly 119 million people worldwide of which more than 2 million have died and the numbers are still rising rapidly. It is considered now a global pandemic disease that threatens public health. SO we need clinical trials to help us find an effective treatment for COVID-19. The study aims to evaluate the effect of atorvastatin on mortality in adult hospitalized patients with COVID-19. This will be done by comparing the mortality rate between the group of patients on atorvastatin and the other group without the drug. Atorvastatin is a drug that was approved for the treatment of hypercholesterolemia and the prevention of other cardiovascular diseases. We expect that it will have a role in the improvement of COVID-19 patients.

1. **The number of people taking part in the study:**

If you agree to participate, you will be one of the about 120 subjects who will be participating in this research study.

1. **Participant selection:**

● Hospitalized patients with confirmed COVID-19 infection who are ≥ 18 years old.

● Disease severity moderate to severe according to WHO clinical progression scale.

● Patient with CK ≤ 4 times the upper limit of normal.

● the patient shouldn’t be a pregnant or lactating women ( confirmed by negative pregnancy test) and on contraception.

● The patient should be able and ready to participate till the end of the study.

● the participant should be able to understand the study and voluntarily sign the informed consent form.

**4) Voluntary Participation:**

Your participation in this research is entirely voluntary. It is your choice whether to participate or not. Whether you choose to participate or not, all the services you receive at this hospital will continue and nothing will change.

**5) Procedures for the study:**

Your consent will be obtained before any procedures.

1- Information about study procedures and requirements will be provided by the physician. Please sign voluntarily the informed consent form if you agree to participate in this study after fully knowing the details of the study.

2- After signing the informed consent, the researcher will record your contact data (your telephone number, telephone number of the relative, your email) and other data (age, sex), any comorbidities (chronic lung disease, hypertension, diabetes mellitus, any cardiac disease, kidney disease, liver disease).

3- The physician will examine you then measure and record blood pressure and oxygen saturation and request lab tests as liver enzymes, serum creatinine, creatine kinase, and CBC.

4-After that, the physician will classify the patient according to the WHO clinical progression scale and ensure that there is no contraindication for the administration of the drug ( pregnancy test should be performed for female patients).

5- If you meet the criteria for registration, you will be randomized to one of two groups in a 1:1 ratio and given an identification number. Randomization means that the group you will be placed in is decided by chance from a computer, similar to drawing numbers out of a hat or flipping a coin.

**6-** You will be assigned to either a group on atorvastatin plus standard treatment or the other group on placebo plus standard treatment.

7- Placebo is an inactive substance containing no study drug, also given orally. We use a placebo because this study is double-blind which means that neither you nor the study doctor will know who is receiving atorvastatin and who is receiving placebo. The placebo will be given the same duration as the drug which is 30 days. There is no adverse effect of using a placebo and you will receive the standard treatment for your case without any delay.

8-we will observe you during hospitalization and make follow up for you after discharge by a phone call on day 30 and 90 from the beginning of the study.

**6) Duration of the study :**

The duration of participation in the study is 90 days.

7) Potential Risks:

A minimal adverse effect may occur as diarrhea, nausea, dyspepsia, arthralgia, and myalgia. This symptom is self relieved without specific treatment. It may cause mild elevation of liver enzymes or diabetes mellitus and we will monitor your lab results for any abnormal changes. There is no risk if you are on the placebo group as you will receive the standard treatment on time.

**8) Potential benefits :**

We will provide you with the drug, or placebo, and all investigations required free of charge. The drug may decrease mortality and other complications of the disease, patients in the placebo group will not receive the same expected benefit. The benefit of the drug is expected but not confirmed. This study may decrease mortality in COVID-19 patients and provide public benefits.

**9) Alternative treatment:**

There are approved therapies for treating COVID-19. Atorvastatin works differently than the approved drugs and we will use it as an additive therapy to the standard treatment to give additional benefits and decrease the mortality rate.

**10) Confidentiality and Sharing the Results:**

All your personal information will be kept confidential. The original data of this study will be preserved by the study authors and the Medical Ethics Committee under the regulations and laws. The results of this study may be published in scientific journals, but no information related to personal identity will be included.

**11) Voluntary withdrawal:**

You may withdraw from the study, at any time, without penalty or loss of benefits to which you are otherwise entitled. Your ongoing medical care will not be affected by your decision.

**12) Contact information:**

If you have questions, concerns, or complaints about the research study, you can contact this No any time:

If you have questions regarding your rights as a research participant, or if you have questions, concerns, complaints about the research contact the person responsible for the [Institutional Research Board - IRB](https://irb.mans.edu.eg/asu6.0/Libraries/IRBsystem.aspx?ScopeID=&BibID=368771) – Mansoura University. Telephone No: 0201092127930.

Local invesitigator contact: Moataz Maher Emara. Telephone No: 0201064048848.

**13) Signing Informed consent form :**

I have read , or it has been read to me, and understood the foregoing information . I have had the opportunity to ask questions about it and any questions that I have asked have been answered to my satisfaction. I consent voluntarily to participate as a participant in this research.

Name of the Participant: ( by the participant or legally authorized representative)

Signature of the Participant: Time: Date:

Signature of legally authorized representative: Time: Date:

**The signing of the witness if any**:

Since the recipient or his guardian was not able to correctly read the content of the informed consent, the researcher will clarify all contents immediately and ensure that the recipient or his guardian understands all the contents of the informed consent and accept to participate in this study . As an impartial witness , I guarantee and confirm all of the prior informed consent form procedures .

Name of the witness:

Contact data of the witness:

Signature of the witness: Time: Date:

I confirm that I have explained all the situations related to the study to the participant . I have provided comprehensive and adequate answers to any questions asked by him (or his guardian) to ensure that the informed consent form has been signed and approved by the participant (or guardian) after a comprehensive explanation and understanding of the study. The participant consent voluntarily , and I will give a copy of the signed informed consent to the participant.

Name of the physician (or researcher ):

Signature of the physician : Time: Date:

**نموذج الموافقة المستنيرة (Arabic Form)**

نموذج الموافقة المستنيرة المبنية على العلم المسبق للدراسة السريرية التي ستجرى على عقار أتورفاستاتين

الرقم التعريفي الخاص بالمشترك في الدراسة:

السيدات و السادة : نأمل ان تكونو بخير و بصحة جيدة! يهدف نموذج الموافقة المستنيرة المبنية على العلم المسبق الى دعوتك للمشاركة في دراسة حول تأثير عقار الأتور على تقليل نسبة الوفيات في المرضى المصابين بفيروس كورونا . بعد ذلك ، سنوضح لك التفاصيل الكاملة للدراسة و الأعراض الجانبية للدواء و فوائدة و غيرها لذا نرجو من سيادتكم قراءة هذة الوثيقة بعناية لمعرفة التفاصيل الكاملة للدراسة و سيتم الاجابة على جميع الاسئلة الخاصة بكم قبل الموافقة على المشاركة في الدراسة.

**1) مقدمة موجزة عن الدراسة :**

سبب فيروس كورونا المستجد و الذي ظهر في الصين في ديسمبر 2019 و اصبح سبب رئيسي للوفاه على مستوى العالم الكثير من المشاكل و المضاعفات على الجهاز التنفسى وأجهزة الجسم المختلفة مما ادى الى زيادة اعداد الوفيات والتي وصلت الى اكثر من 2 مليون حالة حتى الأن . و أدرجت منظمة الصحة العالمية هذة الجائحة كحالة طوارئ تهدد الصحة العامة و تثير القلق على الصعيد الدولي . و نظرا لقلة المعلومات عن هذا الفيروس و قلة الأدوية التي اثبتت فعالية في علاجة و تقليل نسبة الوفيات الناتجة عنة سوف نقوم بهذة الدراسة لمعرفة تأثير عقار الاتور على تقليل نسبة الوفيات في مرضى كورونا و ذلك عن طريق اعطاء مجموعة من المرضى العقار بالاضافة الى العلاج الاساسي و المجموعة الأخرى العلاج الاساسي فقط و تسجيل نسبة الوفيات و درجة تحسن المرضى في كل مجموعة. يعتبر عقار الاتور من الادوية الأمنة و التي تم اعتمادها في علاج ارتفاع نسبة الكوليسترول في الدم و بعض الامراض الاخرى . تتم هذة الدراسة وفقا للوائح الخاصة بجمهورية مصر العربية . تمت مراجعة هذا البحث و الموافقة عليه من قبل لجنة الاخلاقيات بوزارة الصحة.

**2) عدد المشاركين في الدراسة :**

من المقرر ادراج 120 متطوع في الدراسة.

**3) شروط المشاركة:**

● أن يكون المشارك من الأشخاص المؤكد اصابتهم بفيروس كورونا و الذين تم حجزهم بالمستشفى الرئيسي بجامعة المنصورة و الذين تبلغ اعمارهم 18 سنة فاكثر.

● ان يكون المريض من الدرجة المتوسطة او الشديدة طبقا لتقييم منظمة الصحة العالمية ( يتاكد الباحثين المسؤلين عن الدراسة من هذا الأمر) .

●ان لا يكون نتيجة تحليل انزيم كيناز الكرياتينين أعلى من اربع اضعاف المعدل الطبيعي (يتاكد الباحثين المسؤلين عن الدراسة من هذا الأمر).

● أن لا تكون المريضة حاملا او تمارس الرضاعة الطبيعية في وقت المشاركة في التجربة السريرية ( يرفق نتيجة سلبية لاختبار الحمل بالبول) .يتم اتخاذ تدابير فعالة لمنع الحمل خلال فترة اعطاء العقار.

● ان يكون المشارك قادرا و مستعدا على استكمال برنامج و خطة الدراسة السريرية المقررة بالكامل.

● أن يكون المشارك قادرا على فهم الاجراءات و التوقيع طوعا على نموذج الموافقة المستنيرة المبنية على العلم المسبق وان يكون قادرا على الالتزام بمتطلبات و خطة الدراسة.

**4) المشاركة الطوعية:**

مشاركتك في هذا البحث تطوعية بالكامل. سواء اخترت المشاركة أم لا ، ستستمر جميع الخدمات التي تتلقاها في هذا المستشفى ولن يتغير شيء.

**5) الاجراءات المتعلقة بالدراسة :**

سيتم الحصول على موافقتك قبل القيام باي اجراءات متعلقة بالدراسة .

1-الموافقة المستنيرة المبنية على العلم المسبق: سيقوم الطبيب بشرح محتويات هذه الدراسة السريرية لك . يرجى التوقيع طوعا على نموذج الموافقة المستنيرة اذا وافقت على المشاركة في هذه الدراسة بعد المعرفة الكلية لتفاصيل الدراسة و متطلباتها .

2-بعد التوقيع على نموذج الموافقة المستنيرة المبنية على العلم المسبق : سيقوم الباحث بتسجيل بيانات الاتصال ( التليفون و تليفون احد اقارب الدرجة الاولى و الايميل ) البيانات الأخرى ( العمر ، الجنس) و الامراض المصاحبة ( الامراض الرئوية المزمنة ، الضغط ، مرض السكري ، أمراض القلب ، امراض الكلى ، أمراض الكبد).

3- بعد ذلك سيقوم الطبيب المعالج باجراء فحص بدني و قياس ضغط الدم و تشبع الاكسجين و طلب اجراء الفحوصات الطبية لك التي تشمل ما يلي : انزيمات الكبد ،انزيمات الكلى و انزيم كاينيز الكرياتينين و صورة دم .

4-سيقوم الطبيب بتحديد درجة المرض طبقا لمقياس منظمة الصحة العالمي و التاكد من عدم وجود موانع طبية عندك لاستخدام العقار ( ينبغي اجراء اختبار للحمل بالبول للمتطوعات من السيدات ممن هن في سن الانجاب).

5-اذا استوفيت شروط التسجيل و المشاركة سيتم ادراجك كمشارك في الدراسة بنمط عشوائي و سوف تعطى الرقم التعريفي الخاص بك كمشارك في الدراسة بشكل تسلسلي. التوزيع العشوائي يعني أن المجموعة التي سيتم وضعك فيها يتم تحديدها بالصدفة من جهاز كمبيوتر ، على غرار سحب الأرقام من قبعة أو قذف عملة معدنية.

6- سيتم اعطاءك عقار الأتور بالاضافة الى العلاج الأساسي او الدواء الوهمي بالاضافة الى العلاج الأساسي وفقا لرقم تعريف المشارك في الدراسة. تبلغ نسبة عدد المرضى الذين سيتم اعطاءهم العقار الوهمي الى عقار الاتورفاستاتين 1:1. سيتم تقسيم المرضى الى مجموعتين و قد تلتحق عشوائيا باي مجموعة من المجموعتين.

7- الدواء الوهمي هو مادة غير فعالة لا تحتوي على دواء الدراسة و يُعطى عن طريق الفم أيضًا. نستخدم دواءً وهميًا لأن هذه الدراسة مزدوجة التعمية مما يعني أنه لن تعرف أنت أو طبيب الدراسة من الذي يتلقى أتورفاستاتين ومن يتلقى الدواء الوهمي. سيتم إعطاء الدواء الوهمي نفس مدة الدواء وهي 30 يومًا. لا يوجد تأثير سلبي لاستخدام الدواء الوهمي وستتلقى العلاج الأساسي لحالتك دون أي تأخير.

8- سيتم متابعة الحالة اثناء تواجدك بالمستشفى و لمد 90 يوم من بداية الدراسة و سيتم التواصل معك عبر الاتصال الهاتفي في الايام 30 و 90 للاستفسار عن حدوث اي اثار جانبية و عن حالتك الصحية.

.

**6) مدة الدراسة** :

تستغرق مدة المشاركة في الدراسة 90 يوم..

**7) مخاطر متعلقة بالدراسة :**

قد تحدث بعض الاعراض الجانبية الطفيفة و التي تشمل غثيان ، اسهال ، عسر الهضم و الام في المفاصل او العضلات . لا تتطلب هذه الاعراض بصورة عامة علاجا فعادة ما تزول هذه الأعراض من تلقاء نفسها ودون الحصول على وصفة طبية او دواء. أيضا قد يسبب ارتفاع بسيط في انزيمات الكبد او نسبة السكر في الدم وسيتتم المتابعة و عمل التحاليل اللازمة اثناء تواجدك بالمستشفى. لا يوجد خطر إذا كنت في مجموعة الدواء الوهمي حيث ستتلقى العلاج الأساسي في الوقت المحدد.

**8) الفوائد و المزايا :**

.سنوفر لك العقار او الدواء الوهمي مجانا و بدون اي رسوم . كما سنقوم بالتكفل بدفع رسوم و مصاريف الاختبارات و الفحوصات المتعلقة بالدراسة و الفحوصات البدنية و الاجراءات المتعلقة بالبحث و الدراسة. قد يقلل الدواء من معدل الوفيات والمضاعفات الأخرى للمرض ، هذه الفائدة غير موجودة في حالة اذا كنت في المجموعة التي ستتلقى العلاج الوهمي،. و تجرى هذه الدراسة لتقييم فعالية العقار و ليس هناك ما يضمن انك ستستفيد بالتاكيد من هذا العقار. اذا قررت المشاركة في هذه الدراسة ، فقد يساعد هذا الاجراء في علاج فيروس كرونا و تقليل نسبة الوفيات.

**9) الأدوية البديلة :**

هناك ادوية معتمدة لعلاج فيروس كرونا و لكن يعمل الاتورفاستاتين بطريقة مختلفة عن الأدوية المعتمدة الأخرى وسنستخدمة كعلاج اضافي للادوية الأساسية لاعطاء نتائج افضل و تقليل نسبة الوفيات.

**10) اتفاقيات الخصوصية و نشر نتائج البحث**:

سيتم الحفاظ على سرية و خصوصية جميع المعلومات الشخصية الخاصة بك . سيتم الاحتفاظ بالبيانات الأصلية لهذه الدراسة من قبل القائمين على الدراسة و لجنة الاخلاقيات الطبية بما يتوافق مع اللوائح و القوانين . قد يتم نشر نتائج هذه الدراسة في المجلات العلمية ، لكن لايتم ادراج اي معلومات متعلقة بالهوية الشخصية .

**11) الانسحاب من الدراسة في منتصفها:**

نعرب عن رغبتنا في قيامك بالمشاركة في الدراسة بالكامل ، و مع ذلك انت تملك مطلق الحرية و الاختيار بالانسحاب في اي وقت. مهما كان قرارك ، فلن تتحمل اي مسؤلية ناجمة عن الانسحاب و لن يؤثر ذلك سلبا على العلاج الطبي الخاص بك و الحقوق التي تتمتع بها ، و لن تلزم بسداد اي رسوم او مبالغ. و بالطبع قد يطلب منك الطبيب الانسحاب من الدراسة في اي وقت و قد يرجع ذلك الى العديد من الأسباب المختلفة ، مثل الخوف على صحتك او عدم اتباعك للتعليمات و الارشادات الخاصة بالدراسة او ايقاف الدراسة نفسها.

**12) معلومات الاتصال و التواصل**:

**اذا كانت لديك أي اسئلة بخصوص الحصول على النصيحة و المساعدة ، يرجى الاتصال على رقم الهاتف التالي في اي وقت**

.

**من اجل الحفاظ على حقوقك و اهتماماتك كمشارك في الدراسة يمكنك التواصل مع:**

مسؤول لجنة اخلاقيات كلية الطب – جامعة المنصورة

الهاتف:

محمد حمادة
irb.mfm@hotmail.com
+20 109 212 7930

الرقم التعريفي الخاص بالمشارك في الدراسة:

رقم تليفون الباحث الرئيسي: 0201064048848

**13) التوقيع على نموذج الموافقة المستنيرة المبنية على العلم المسبق:**

يرجى قراءة المربع التالي

لقد قمت بقراءة و فهم جميع ما سبق بالتفصيل ، كما فهمت و وافقت على جميع متطلبات المشاركة في الداسة ، حيث قام الباحث بايضاح الأمر تفصيلا و الاجابة على جميع أسئلتي ؛ و بعد الالمام بكامل الأمر و التفكير الكافي ، أوافق على مشاركتي في الدراسة بشكل تطوعي و التوقيع باسمي في نموذج الموافقة المستنيرة المبنية على العلم المسبق.

اسم المتطوع ( بخط واضح) : (يتم تسجيلة من قبل المتطوع / الممثل المقبول قانونا).

توقيع المتطوع : الوقت : التاريخ: (..../...../.......) يوم/ شهر/ سنه.

توقيع الممثل المقبول قانونا : الوقت : التاريخ : (..../...../......) يوم / شهر / سنه.

توقبع الشاهد المحايد (في حالة وجود شاهد محايد) يرجى قراءة المربع التالي :

نظرا لأن المتلقي او الوصي عليه لم يتمكن من قراءة محتوى الموافقة المستنيرة المبنية على العلم المسبق بشكل صحيح ، فسيقوم الباحث بايضاح جميع المحتويات على الفور و الحرص على فهم المتلقي او الوصي عليه لجميع محتويات الموافقة المستنيرة المبنية على العلم المسبق و الموافقة على المشاركة في هذه الدراسة . كشاهد محايد اقر و اشهد على جميع اجراءات نموذج الموافقة المستنيرة المبنية على العلم المسبق بأكملها.

اسم الشاهد المحايد (بخط واضح ) :

بيانات التواصل مع الشاهد :

توقيع الشاهد المحايد: الوقت : التاريخ: (..../..../.....) يوم/ شهر/ سنه.

أقر أنني قد قمت بتوضيح جميع المواقف و الحالات المتعلقة بالدراسة التي ستجرى على المتطوع. لقد قمت بتقديم اجابات شاملة و كافية على اي اسئلة قام بطرحها المتطوع (او الوصي عليه) للتاكد من ان نموذج الموافقة المستنيرة المبنية على العلم المسبق قد تم توقيعة و الموافقة عليه من قبل الشخص المتطوع ( او الوصي عليه) بعد التوضيح و الفهم بشكل شامل للدراسة و مع منحهم حريه الاختيار و ساقوم بتسليم نسخة الموافقة المستنيرة المبنية على العلم المسبق الموقعة الى المتطوع ( او الوصي عليه)

اسم طبيب الدراسة:

توقيع طبيب الدراسة: الوقت: التاريخ: (..../...../.......) يوم/ شهر/ سنه.
