# Supplementary material for: Atorvastatin for reduction of 28-day mortality in hospitalized COVID-19 patients: study protocol for a randomized, double-blinded, placebo-controlled, clinical trial
Source: Trials. 2022 Aug 8;23:636. doi: 10.1186/s13063-022-06619-9 (PMC9360729; doi:10.1186/s13063-022-06619-9)
Supplement: Supplementary file 3 — Additional file 3: Supplement S3. Case Report File (CRF) [file 13063_2022_6619_MOESM3_ESM.docx]

**Atorvastatin for Reduction of 28-day Mortality in Adult Hospitalized Patients with COVID-19: A Randomized Clinical Trial**

**Case Report File (CRF)**

**PATIENT DATA*:**

**Patient Name:**

**National ID: ☐☐☐☐☐☐☐☐☐☐☐☐☐☐**

**Date of admission: DD-MM-YYYY**

**Telephone No:**

**Alternative telephone No:**

**Assigned group:**

1. **All items are to be fulfilled with a black or dark blue ballpoint pen**
2. **The Investigator MUST write the patient ID on each page**
3. **The Investigator MUST write his name clearly down at least at the bottom of the first page**
4. **In case of error, draw a single line through the error, so the original entry is visible, and write the correct value next to it. Date and sign the correction.**
5. **Selections with square boxes (☐) are single selection answers (choose one answer only).**
6. **Selections with circular boxes (◯) are multiple selection answers (choose all that apply).**

**Inclusion Criteria*:**

**(All items must be answered with YES, otherwise the subject is not eligible for the study)**

1. Age ≥ 18 years. ☐ Yes (1) ☐ No (0)
2. Diagnosed clinically , radiologically or PCR confirmed

COVID-19 infection. ☐ Yes (1) ☐ No (0)

1. Sever or critical COVID 19 acc.to WHO. ☐ Yes (1) ☐ No (0)
2. Written Informed Consent. ☐ Yes (1) ☐ No (0)

**Exclusion Criteria*:**

**(All items must be answered with NO, otherwise the subject is not eligible for the study)**

1. Prior statin use. ☐ Yes (1) ☐ No (0)
2. Serum creatine kinase> 5 times the upper limit of normal (ULN) ☐ Yes (1) ☐ No (0)
3. Serum transaminases > 5 times the upper limit of normal (ULN) ☐ Yes (1) ☐ No (0)
4. Acute hepatic failure. ☐ Yes (1) ☐ No (0)
5. Chronic liver disease child-Paugh Classification C. □ Yes(1) □ No (0)
6. History of rhabdomyolysis or myopathies. □ Yes(1) □ NO(0)
7. Sever renal impairment not receiving renal replacement therapy

(estimated CrCl< 30 ml/min) □ Yes (1) □ NO (0)

1. Pregnant or lactating women. □ Yes (1) □ NO (0)
2. Treatment with cyclosporine , gemfibrozil , fusidic acid (systemic )

or anti-hepaciviral (Ombitasvir, paritaprevir , ritonavir). □ Yes (1) □ NO (0)

1. Patient on chronic colchicine. □ Yes (1) □ NO (0)
2. Patient expected to die within 48 hours. □ Yes (1) □ NO (0)

**PATIENT DATA:**

**Patient Name*:**

**National ID*: ☐☐☐☐☐☐☐☐☐☐☐☐☐☐**

**Date of admission*:** DD-MM-YYYY

**Assigned group*:**

**Patient age*:** …………. (Years) the difference between the date of admission and the date of birth

**Sex*:** ☐ M (1) ☐ F (2)

**Weight:** --- kg

**Height:** ---- m (2 decimals)

**Smoking**: □Never (0)- □Current(1)- □Former(2)- □Unknown(3).

**Substance’s abuse:** □ Yes(1)-. □ No(0). If yes (□ alcohol- □ drug -□ other)

**Healthcare worker:** □ Yes(1)-. □ No(0).

**Laboratory worker:** □ Yes(1)-. □ No(0).

**MEDICAL HISTORY:**

1. **Cardiac disease:** ☐ Yes (1) ☐ No (0)

If yes, duration ………..years

1. **Hypertension:** ☐ Yes (1) ☐ No (0)

If Yes, duration ……..years

1. **Chronic Liver disease:** ☐ Yes (1) ☐ No (0)

If yes, duration…………. Years

Child-Pugh score:

1. **Diabetes Mellitus:** ☐ No (0) ☐ NIDDM (1) ☐ IDDM (2)

If yes, duration ………..years

1. **Chronic Kidney Disease:** ☐ Yes (1) ☐ No (0)

If yes, duration ………..years

1. **Dialysis:** ☐ Yes (1) ☐ No (0)

If yes, duration ………..years

1. **Chronic Pulmonary disease:** ☐ No (0) ☐ COPD (1) ☐ B.A (2)

If others………… if yes, duration……… years.

1. **Cancer:** ☐ Yes (1) ☐ No (0)

If yes, onset……….year

1. **Chronic neurological disorder:** ☐ Yes (1) ☐ No (0)

If yes duration or onset ………years.

1. **Mental health condition:** ☐ Yes (1) ☐ No (0)
2. **Previous COVID infection.** ☐ Yes (1) ☐ No (0)
3. **Vaccination status for COVID**. ☐ No (0) ☐Astrazeneca one dose (1). ☐ Astrazeneca two doses(2). ☐Sinopharm one dose (3). ☐ Sinopharm two doses (4). ☐ Others (5).
4. **Others** ………………………..

**ON ADMISSION**

**Date of admission:** DD-MM-YYYY.

1. **Diagnosis**:

**Date of diagnosis:** DD-MM-YYYY.

**Diagnosis*:** ☐ symptoms + doctor suspicion only (1).

☐ Radiology and Lab (2).

☐ PCR confirmed (3).

**Severity of COVID-19*:** □ sever COVID 19 infection.

If sever (◯ SpO2 <90% on room air- ◯RR > 30 breaths/min)

□ critical COVID 19 infection.

If critical infection (◯ ARDS- ◯ sepsis - ◯septic shock –

◯ pulmonary embolism- ◯Acute coronary syndrome -◯Acute stroke)

**WHO clinical progression score*:** …………

1. **Symptoms:**

**Symptoms onset** : DD-MM-YYYY

**Fever:** ☐ Yes (1) ☐ No (0)

**Respiratory symptoms:** ☐ Yes (1) ☐ No (0)

If yes (◯ Cough - ◯sore throat -◯ Runny nose- ◯shortness of breath-◯ dyspnea - ◯wheezing- ◯ chest pain )

**Gastrointestinal symptoms:** ☐ Yes (1) ☐ No (0)

If yes (◯ Diarrhea- ◯ nausea or Vomiting- ◯abdominal pain)

**Loss of taste or smell:** ☐ Yes (1) ☐ No (0)

If yes ( ◯ Loss of taste- ◯ Loss of smell ).

**Neurologic symptoms:**

(☐ No (0) ☐ Seizures (1)- ☐Altered consciousness/confusion (2)).

**General symptoms:** ☐ Yes (1) ☐ No (0)

If yes (◯ Headache- ◯muscle aches (myalgia) – ◯joint pain (arthralgia)- ◯Red . swollen eye- ◯Skin rashes-◯fatigue- ◯inability to walk- ◯ others)

If others……………

1. **Vital signs on admission* :**

**Bp : ………. HR:………. Temp: ……… RR:……….**

**Oxygen saturation: ………..** On □ Room air (0) . □Oxygen (1).

1. **Labs and Scores on admission :**

| **parameter** | **value** | **Not done** | **parameter** | **value** | **Not done** | **parameter** | **value** | **Not done** |
| --- | --- | --- | --- | --- | --- | --- | --- | --- |
| ***AST**** |  | **□** | ***TLC* (x10^9^/L)** |  | **□** | **procalcitonin** |  | **□** |
| ***ALT**** |  | **□** | ***Lymphocytes*** |  | **□** | **sodium** |  | **□** |
| ***Albumin**** |  | **□** | ***Hb* (g/L)** |  | **□** | ***potassium*** |  | **□** |
| ***Bilirubin**** |  | **□** | ***Haematocrit*** |  | **□** | ***PaO_2_*** |  | **□** |
| ***PT**** |  | **□** | ***PLT*** |  | **□** | **PaCO_2_** |  | **□** |
| ***INR*** |  | **□** | ***LDH*** |  | **□** | **PaO2/Fio2** |  | **□** |
| ***FBG*** |  | **□** | ***D-dimer*** |  | **□** | **GCS Score** |  | **□** |
| ***SrCr**** |  | **□** | ***CRP*** |  | **□** | **SOFA Score** |  | **□** |
| ***CK**** |  | **□** | ***Ferritin*** |  | **□** |  |  |  |

1. **Blood Culture ☐ done ☐ Not done .**

**If done , The organism …………….**

**Sensitive to ……………….**

**DURING HOSPITALIZATION**

1. **Treatment:**

**1-Antibiotics *** ☐ Yes (1) ☐ No (0).

If yes (□ Macrolide - □Fluroquinolones -□ 3^rd^ and 4^th^ cephalosporin-□Carbapenems- □ Piperacillin+tazobactam- □Amoxicillin+ clavulonate - □ Cotrimoxazole - □ others)

● Macrolide (□No (0)- □ azithromycin (1)- □ clarithromycin (2))

● Fluroquinolones (□No (0) -□ Ciprofloxacin (1) - □ levofloxacin (2)- □ moxifloxacin(3)- □ciprofloxacin + levofloxacin (4))

● 3rd and 4th cephalosporin (□No (0)- □Ceftriaxone (1) - □Cefotaxime (2) -□ ceftazidime (3) - □ cefepime(4))

● Carbapenems (□No (0)- □ imipenem(1) - □ meropenem (2))

- Linezolid (□No (0)- □ yes(1) )
- Teicoplnin ( □ No (0)- □ yes (1) )

● others ………….

Started on: DD-MM-YYYY. Stopped on: DD-MM-YYYY

**2- anticoagulant drugs.**

(□No (0). □UFH (1) - □ enoxaparin (2) -□ fondaparinux (3) □warfarin (4) - □ dabigatran (5) - □ rivaroxaban (6) - □abixaban (7) - □ endoxaban (8)- enoxaparin+ rivaroxaban (9)- recombinant hirudin (10)- UFH+ recombinant hirudin (11)).

Dose ( □theraputic (1) - □ preventive (2))

Started on : DD-MM-YYYY. Stopped on : DD-MM-YYYY

**Antiplatelet** (□No(0) -□aspirin (1)- □ clopidogrel (2) - □ ticagrelor (3)- aspirin+clopidogrel (4) )

**3- Antiviral drug** .

( No (0)-□lopinavir/ritonavir (1) - □ remdesivir(2) - □ oseltamivir(3) -□favipiravir (4) - □ remdesivir + favipravir (6)- □ ribvirin (7)- □ribavirin+remdesivir (8)- □ribavirin+ fvipravir (9) - □remdesivir + favipiravir + ribavirin (10)) )

Started on : DD-MM-YYYY. Stopped on : DD-MM-YYYY

**4- Blood-derived products**

(□No (0)- □ IV immunoglobulin (1) - □ Convalescent plasma (2)- □ albumin(3)- □RBCs (4)- □RBCs+albumin (5)- □platelet (6) ).

If others,………….

Started on : DD-MM-YYYY. Stopped on : DD-MM-YYYY

**5-Steroid received.**

(□No(0) –dexamethasone (1) - □hydrocortisone (2)- □prednisone (3) -□methylprednisolone (4) - □dexamethasone + methylprednisolone (5) )

Route ( □ Oral - □ intravenous - □ inhaled) Dose:…………

Started on : DD-MM-YYYY. Stopped on : DD-MM-YYYY

**6-Chloroquine / hydroxychloroquine**. ☐ Yes (1) ☐ No (0)

Started on : DD-MM-YYYY. Stopped on : DD-MM-YYYY

**7-Ivermectin** ☐ Yes (1) ☐ No (0)

Started on : DD-MM-YYYY. Stopped on : DD-MM-YYYY

**8-interferon** ☐ Yes (1) ☐ No (0)

Started on : DD-MM-YYYY. Stopped on : DD-MM-YYYY

**9-Eculizumab** ☐ Yes (1) ☐ No (0)

Started on : DD-MM-YYYY. Stopped on : DD-MM-YYYY

**10-IL-6 antagonist (Tocilizumab)** ☐ Yes (1) ☐ No (0)

Started on : DD-MM-YYYY. Stopped on : DD-MM-YYYY

**11-Kinas Inhibitors (barcitinib).** ☐ Yes (1) ☐ No (0)

Started on : DD-MM-YYYY. Stopped on : DD-MM-YYYY

**12- additive therapy** ☐ Yes (1) ☐ No (0)

If yes, (□paracetamol-□Zinc - □ acetylcysteine- □lactoferrine – □vitamin C- □others).

If others………….

Started on : DD-MM-YYYY. Stopped on : DD-MM-YYYY

**13-others**,……………….

Started on : DD-MM-YYYY. Stopped on : DD-MM-YYYY

1. **Supportive care :**
2. **Oxygen therapy? *** ☐ Yes (1) ☐ No (0)

If yes, O2 flow (□ 1-5 L/min - □6-10 L/min- □ 11-15 L/min-□ >15 L/min-□ Unknown)

Interface (□ Nasal prongs□ HF nasal cannula-□ Mask-□ Mask with reservoir - □CPAP/NIV mask)

Started on : DD-MM-YYYY. Stopped on : DD-MM-YYYY

1. **Non-invasive ventilation? (e.g. BIPAP/CPAP)*** ☐ Yes (1) ☐ No (0)

Started on: DD-MM-YYYY. Stopped on : DD-MM-YYYY

1. **Invasive ventilation (Any)?** * □ Yes (1) □No (0)

Started on: DD-MM-YYYY. Stopped on: DD-MM-YYYY

1. **Inotropes/vasopressors? *** ☐ Yes (1) ☐ No (0)

Started on: DD-MM-YYYY. Stopped on: DD-MM-YYYY

1. **Prone position?** ☐ Yes (1) ☐ No (0)
2. **Labs & Scores & Vital signs*:**

| **Test** | **Day 1** | ***Day 3*** | ***Day 7*** | ***Day 14*** | ***Day 28*** | ***As needed***  ***(Write date)*** |
| --- | --- | --- | --- | --- | --- | --- |
| **AST** |  |  |  |  |  |  |
| **ALT** |  |  |  |  |  |  |
| **Billirubin** |  |  |  |  |  |  |
| **SrCr** |  |  |  |  |  |  |
| **CK** |  |  |  |  |  |  |
| **CBC (PLT)** |  |  |  |  |  |  |
| **FBG** |  |  |  |  |  |  |
| **PaO_2_/FiO_2_** |  |  |  |  |  |  |
| **BP** |  |  |  |  |  |  |
| **D-dimer** |  |  |  |  |  |  |
| **CRP** |  |  |  |  |  |  |
| ***GCS*** |  |  |  |  |  |  |
| ***SOFA score*** |  |  |  |  |  |  |

| **WHO Scale** | ***Day 1*** | ***Day 2*** | ***Day 3*** | ***Day 4*** | ***Day 5*** | ***Day 6*** |
| --- | --- | --- | --- | --- | --- | --- |
|  |  |  |  |  |  |  |
|  | ***Day 7*** | ***Day 8*** | ***Day 9*** | ***Day 10*** | ***Day 11*** | ***Day 12*** |
|  |  |  |  |  |  |  |
|  | ***Day 13*** | ***Day 14*** | ***Day 15*** | ***Day 16*** | ***Day 17*** | ***Day 18*** |
|  |  |  |  |  |  |  |
|  | ***Day 19*** | ***Day 20*** | ***Day 21*** | ***Day 22*** | ***Day 23*** | ***Day 24*** |
|  |  |  |  |  |  |  |
|  | ***Day 25*** | ***Day 26*** | ***Day 27*** | ***Day 28*** |  | |
|  |  |  |  |  |  |  |

***OUTCOMES****

1. 28 -day mortality. ☐ Died (1) ☐ Not died (0)
2. Need for invasive mechanical ventilation □ Yes (1) □ NO (0)
3. Invasive mechanical ventilation and oxygen support duration (days)………….
4. Time to clinical improvement (days) ………..

(2 points reduction in the WHO disease ordinal progression scale or discharge whatever happens first).

1. Serious adverse effect leading to drug discontinuation. □ Yes (1) □ NO (0)

If yes , ……………….

1. Length of hospital stay (days) ……..
2. Length of ICU stay (days ) ………..
3. CRP ( Day 1 ………. Day 3………. Day 7………… Day 14……….. Day 28……….)
4. SOFA score (Day 1 …… Day 3……. Day 7……….. Day 14………. Day 28……….)
5. COVID 19 WHO disease progression score .

( Day 1 ……. Day 3…….. Day 7……. Day 14 …… Day 28………).

1. Incidence of AKI. □ Yes (1) □ NO (0)

12-mortality at hospital discharge. □ died (1) □ Not died (0)

13-Inhospital DVT. □ Yes (1) □ NO (0)

14-Inhospital PE. □ Yes (1) □ NO (0)

15- D-dimer (Day 1…….. Day 7…….. Day 14……… Day 28……..).

**SCORES**

**Child-paugh score:**

|  | **1 point** | **2 points** | **3 points** |
| --- | --- | --- | --- |
| **Ascitis** | **Absent** | **Slight** | **moderate** |
| **Billirubin ( mg/dl)** | **< 2** | **2-3** | **> 3** |
| **Albumin (g/dl)** | **>3.5** | **2.8-3.5** | **<2.8** |
| **PT ( sec. over control)**  **Or INR** | **<4**  **<1.7** | **4-6**  **1.7-2.3** | **>6**  **>2.3** |
| **Encephalopathy** | **None** | **Grade 1-2** | **Grade 3-4** |

Score 5-6 is considered class A 7-9 is Class B 10-15 is class C

| Patient State | Descriptor | Score |
| --- | --- | --- |
| Uninfected | -Uninfected, no viral RNA detected. | 0 |
| Ambulatory mild disease | -Asymptomatic; viral RNA detected.  -Symptomatic; independent.  -Symptomatic; assistance needed. | 1  2  3 |
| Hospitalised: moderate disease | Hospitalized; no oxygen therapy.  Hospitalized; oxygen by mask or nasal prongs. | 4  5 |
| Hospitalized: sever disease | -Hospitalized; oxygen by NIV or high flow.  -Intubation and mechanical ventilation, pO_2_ /FIO_2_  ≥ 150 or Spo_2_ /FiO_2_ ≥200.  -Mechanical ventilation pO_2_/FiO_2_ <150 (SpO_2_ /FiO_2_ < 200) or vasopressors.  -Mechanical ventilation pO_2_ / FiO_2_ < 150 and vasopressors, dialysis or ECMO. | 6  7  8  9 |
| Dead | Dead | 10 |

**WHO clinical progression scale**

**Glasgow coma score (GCS):**

| **Response** | **Score** | **Scale** |
| --- | --- | --- |
| **Eye opening response** | **spontaneous** | **4** |
|  | **To verbal command** | **3** |
|  | **To pain** | **2** |
|  | **None** | **1** |
| **Verbal response** | **Oriented conversation** | **5** |
|  | **Disoriented conversation** | **4** |
|  | **Inappropriate words**  **Incomprehensible words** | **3** |
|  | **Incomprehensible sound** | **2** |
|  | **None** | **1** |
| **Motor response** | **Obeys verbal command** | **6** |
|  | **Localized painful stimuli** | **5** |
|  | **Flexion withdrawal from painful stimuli** | **4** |
|  | **Decorticate response to painful stimuli** | **3** |
|  | **None** | **1** |

**SOFA score:**

| **variables** | **0** | **1** | **2** | **3** | **4** |
| --- | --- | --- | --- | --- | --- |
| **Respiratory**  **PaO_2_/FiO_2_** | > 400 | ≤ 400 | ≤ 300 | ≤ 200 with respiratory support | ≤ 100 with respiratory support |
| **Hematologic**  **PLT** | >150  ×10^3^/mm^3^ | 101-150  ×10^3^/mm3 | 51-100  ×10^3^/mm3 | 21-50  ×10^3^/mm3 | ≤20  ×10^3^/mm3 |
| **Hepatic**  **bilirubin** | <1.2 mg/dl | 1.2-1.9 mg/dl | 2-5.9 mg/dl | 6-11.9 mg/dl | >12 mg/dl |
| **Blood Pressure** | Hypotension absent | MAP< 70 mmHg | On dopamine≤ 5 mcg/Kg/min or any dobutamine | On dopamine > 5 mcg/ kg/ min , Epinephrin≤ .1 mcg/kg/min or norepinephrine ≤ .1 mcg/ kg/ min | On dopamine >15 mcg/ kg/ min or epinephrine >0.1 mck/kg/min or norepinephrine> .1 mcg/kg/min |
| **Brain (GCS)** | 15 | 13-14 | 10-12 | 6-9 | <6 |
| **Kidney**  **Renal function** | Creatinine < 1.2 mg/dl | Creatinine 1.2-1.9 mg/dl | Creatinine 2-3.4 mg/dl | Creatinine 3.5-4.9 mg/dl or UOP 200-500 ml/day. | Creatinine >5 mg/dl or UOP <200 ml/day |

**DEFINITIONS**

**Sever COVID 19 infection:** have the clinical signs of severe pneumonia and SpO2 <90% on room air, or RR > 30 breaths/min without any critical criteria

**Critical COVID 19 infection (WHO):** have ARDS, sepsis, septic shock, pulmonary embolism, acute coronary syndrome, or acute stroke

**Acute liver failure:** evidence of coagulopathy, usually considered an INR of 1.5 or greater, and any degree of mental alteration (encephalopathy) in a patient without preexisting liver failure and with an illness duration less than 26 weeks.

**Acute kidney injury:** increase in SrCr by ≥ .3 mg/dl within 48 hr or increase in SrCr by ≥ 50% in 7 days or oliguria for ≥ 6 hrs.

**Sepsis:** Life-threatening organ dysfunction caused by a dysregulated host response to infection ( Organ dysfunction can be identified as an increase of 2 points or more in SOFA score ).

**Sepsis (WHO):** Life-threatening organ dysfunction caused by a dysregulated host response to infection. Signs of organ dysfunction include : altered mental status ( delirium ), difficult or fast breathing , low oxygen saturation , reduced urine output , fast HR, weak pulse, cold extremities or low blood pressure , skin mottling, laboratory evidence of coagulopathy , thrombocytopenia , acidosis , high lactate or hyperbilirubinemia.

**Septic shock:** sepsis with persistent hypotension despite volume resuscitation, requiring vasopressor to maintain a MAP ≥ 65 mmHg and serum lactate level > 2 mmol/l.
