# Supplementary material for: Atorvastatin for reduction of 28-day mortality in hospitalized COVID-19 patients: study protocol for a randomized, double-blinded, placebo-controlled, clinical trial
Source: Trials. 2022 Aug 8;23:636. doi: 10.1186/s13063-022-06619-9 (PMC9360729; doi:10.1186/s13063-022-06619-9)
Supplement: Supplementary file 1 — Additional file 1: Supplement S1. IRB approval [file 13063_2022_6619_MOESM1_ESM.pdf]

Medical Research Ethics Committee
Institutional Review Board
Mansoura Faculty Of Medicine
Mansoura University

Acceptance Letter

Chairman
Ashraf Sewelam
Co-Chairman
Tarek El-Diasty
Coordinator
Sameh Emile
Members
salah Elgamal
Reda Hemida
Ibtihal M.Ali
Eman Khashaba
Ali Sobh
Hossam Elfeki
Tamer Saleh
Tarek Abdl hady
Adminstrator
Mohamed Hamada

Title: Efficacy of Atorvastatin in Severe and Critically-ill Patients with COVID-19: A Randomized, Double-blinded, Placebo-Controlled, Clinical Trial

Main Author (Single): Moataz Maher Emara (Moataz M. Emara)Lecturer of Anaesthesiology and Intensive Care , Faculty of Medicine, Mansoura University

Code Number: R.21.04.1300

Date: 2 /06/2021

Dear Dr: Moataz Maher Emara

I am pleased to let you know that your work has now been accepted by IRB of Mansoura Faculty of Medicine, Mansoura University.

Looking Forward to receiving your future work.

Please feel free to contact us for any request.

With Kind Regards
Prof.Dr.Ashraf Sewelam
Ashraf Sewelam
Professor of Ophthalmology
Former vice President of Mansoura University
Chairman of the Ophthalmic Scientific Committee
Chairman of Medical Research Ethics Committee
Faculty of Medicine - Mansoura University
8/6/2021

Address : Institutional Review Board (IRB) Office Building A-Ground Floor - Faculty of Medicine - Mansoura University
Email : IRB.MFM@hotmail.com **Website** : http://irb.mans.edu.eg
Mob.: +201092127930 **Phone#** : +2 (050) 2202773 - 2202746

Scanned by TapScanner
